# Bed Bugs: The Australian Response

**DOI:** 10.3390/insects2020096

**Published:** 2011-04-15

**Authors:** Stephen L. Doggett, Christopher J. Orton, David G. Lilly, Richard C. Russell

**Affiliations:** 1 Department of Medical Entomology, ICPMR, Westmead Hospital, Westmead, NSW, 2145, Australia; 2 School of Biological, Earth and Environmental Sciences, Faculty of Science, University of New South Wales, Sydney, NSW, 2052, Australia; E-Mail: c.orton@unsw.edu.au; 3 Ecolab Pty Ltd, Pest Elimination Division, 6 Hudson Ave, Castle Hill, NSW, 2154, Australia; E-Mail: David.Lilly@ecolab.com; 4 Department of Medical Entomology, University of Sydney at Westmead Hospital, Westmead, NSW, 2145, Australia; E-Mail: richard.russell@sydney.edu.au

**Keywords:** Australia, bed bugs, *Cimex*, management

## Abstract

Australia has experienced a sudden and unexpected resurgence in bed bug infestations from both *Cimex lectularius* L. and *Cimex hemipterus* F. A survey in 2006 revealed that infestations had increased across the nation by an average of 4,500% since the start of the decade. In response, a multi-disciplinary approach to combat the rise of this public health pest was implemented and involved the coordinated efforts of several organizations. The key components of the strategy included the introduction of a pest management standard ‘A Code of Practice for the Control of Bed Bug Infestations in Australia’ that defines and promotes ‘best practice’ in bed bug eradication, the development of a policy and procedural guide for accommodation providers, education of stakeholders in best management practices, and research. These strategies continue to evolve with developments that lead to improvements in ‘best practice’ while bed bugs remain problematic in Australia.

## 1. Introduction

In just over a decade, a pest that had virtually disappeared has made an astonishing come back. It is not only the degree of the resurgence that has shocked and surprised many, but also the variety of places that bed bugs have come to infest and the widespread geographic spread of the insect. The return of the bed bug has been recorded across Europe [1–4], Asia [4–8] and the Americas [4,9]. Australia has not been excluded [10,11], but unlike many other countries, the resurgence has involved both the common (*Cimex lectularius* L.) and tropical (*Cimex hemipterus* F.) bed bug species [11,12].

It was apparent quite early in the resurgence that no ‘silver bullet’ for bed bug management was likely to be developed in the foreseeable future, and that no single approach was likely to succeed in the long term defeat of this nuisance pest. Thus a multi-disciplinary strategy was implemented and the key components included: the introduction of a pest management standard called ‘A Code of Practice for the Control of Bed Bug Infestations in Australia’ that promotes ‘best practice’ in the management of bed bug infestations [13], the development of a policy and procedural guide for accommodation providers [14], education of stakeholders in best management practices, and research. These strategies have co-evolved, for example the development of the Code of Practice highlighted deficiencies in our knowledge on many aspects of bed bug management. This subsequently led to research investigations whereby the results were then used to further develop the industry standard.

The development of these strategies has come about through the cooperation of the peak pest management industry association (the Australian Environmental Pest Managers Association), pest controllers, insecticide manufacturers, health workers and entomologists. This paper reviews the history of bed bug activity in Australia, the need for an industry standard and its developmental processes, the educational activities undertaken for the various stakeholders, and bed bug research undertaken within the country.

## 2. Bed Bugs in Australia

The history of bed bugs in Australia has been previously documented [11]. Briefly, indications are that bed bugs (presumably the common species) were first brought into the country with the early European colonialists in the 18th century via sailing vessels and soon became a widespread, yet unwanted, cohabitant of urban dwellings. Even well into the 20th Century bed bugs were such a regular part of everyday life that the eminent entomological text of the mid-1920s refused to discuss the insects in any details, stating: “[the Cimicidae is] represented [in Australia] by the detestable and all too common…bed bug…which is too well known to need description here…” [15].

As elsewhere, the decline of bed bugs that occurred post World War II in Australia was believed to be due to the introduction, availability and widespread use of the organochlorine insecticides. However, there is little documentary history of this event.

The first evidence of a bed bug resurgence was provided in 2004 [10]. It was reported that the insect pathology service at Westmead Hospital (the laboratory for authors SLD and RCR) saw an increase of the order of 250% in the number of bed bugs being submitted between 2001–2004, compared with the previous four years. One pest management company had noted an increase in bed bug treatments of around 700%, while other government agencies also observed an upturn in bed bug enquiries or specimens. Soon after, a report was released of a bed bug survey of short stay accommodation within Sydney [16]. It was documented that by 2003, 79% of such facilities had recently experienced bed bug infestations.

Early in the new millennium the first record of the tropical bed bug (*C. hemipterus*) was made in the country [12]. Subsequently, this species has become widespread through regions north of roughly the 29° S latitude [11]. Occasional infestations occur outside this range and are typically associated with travelers from tropical regions. More recent investigations of preserved specimens from various medical entomology laboratories have revealed that the tropical bed bug has been present within Australia since at least the early 1990s [11]. In relation to the modern bed bug resurgence, it appears that the impact of the tropical species was felt much earlier than the common bed bug, which is perhaps not surprising considering that bed bug life cycles are shorter in warmer climates [17]. For example, in 1999 one popular tropical resort undertook 370 separate bed bug treatments in their staff accommodation block, which were all presumably due to tropical bed bug infestations as the resort is located within the region of this species distribution. The first bed bug forum to be held anywhere in the world associated with the modern resurgence was conducted over two days in 2004 (13–14 September) at Airlie Beach in Northern Queensland, and again this was due to tropical bed bug activity.

It is often forgotten that the resurgence involves two species, and not just the common bed bug, which makes the exact determination of the causes of the resurgence more challenging to ascertain. Elsewhere worldwide, other than very recent publications of infestations in south-east Asia [6,7], there have been few reports of the reappearance of the tropical bed bug. Anecdotal experiences of the authors, suggest other tropical nations are also experiencing a resurgence. Nearly all infestations of *C. hemipterus* in southern Australia are believed to have been brought in via students from the Indian subcontinent. For most tropical regions there is less wealth and consequently poorer health infrastructures, and such areas have higher entomological priorities in having potentially fatal endemic vector-borne diseases such as malaria and dengue to combat. In light of bed bugs having relatively minor health impacts, it is little wonder that these insects are often ignored in such regions.

In 2006 a comprehensive survey of Australian pest managers was undertaken to better ascertain the degree of the resurgence and the type of properties being treated [11]. Bed bug management procedures were also surveyed in order to address education of pest managers in a bed bug industry standard that was in the process of development. The survey revealed that infestations had risen across all states and overall for the nation by 4,500% for the period of 1999 to June 2006, compared with pre-1999 levels. Most infestations (∼46%) occurred in either 1–3 star motels or backpacker lodges. The survey of treatment methods noted that a number of procedures were being undertaken with little value (e.g., using black plastic for thermal control of infested mattresses), numerous insecticide products not registered for use against bed bugs were being regularly used, and follow up inspections after initial treatments often were not undertaken. This all highlighted the need for better education of pest managers.

One interesting aspect of the above survey was that there were very few reports of bed bugs occurring in low income housing. It appears that the socially disadvantaged were not impacted heavily until around the middle years of the first decade in the new millennium. It was in early 2006 that massive infestations involving tens to hundreds of thousands of bed bugs began to be reported to the authors. These were typically occurring amongst socially disadvantaged groups where the residents did not have the financial resources to pay for control or, in some instances, the cognitive awareness to recognize the presence of an infestation. By 2009, several of the authors were on task forces to address the problem of bed bugs in low income housing. One comprehensive investigation led to the development of a management policy for one community housing group [18]. This was later to form the basis of the management policy for accommodation providers discussed below.

The survey from 2006 was able to provide an approximation of the economic consequences of the bed bug resurgence to the nation. Based on detailed recording of the costs associated with bed bug eradication in a staff accommodation facility and a three star hotel, it was estimated that the cost to the nation in 2006 was in the order of AUS$100million (see reference [11] for detailed calculations of this amount). By the start of 2011, this amount could easily have doubled. Naturally, such calculations are an extremely conservative estimate and do not include financial impacts such as litigation, societal control (such as the development of industry standards, procedural guides, training and education), or potential future loss of earnings through reduced productivity of persons with infestations or declining patronage of accommodation providers with damaged reputations. Nor can such figures calculate the human morbidity or mental trauma that is often associated with bed bug infestations. It is clear that while bed bugs remain problematic, the associated costs will continue to escalate. The accurate determination of the financial impacts of bed bugs is an imperative and may help to encourage government and granting bodies to provide the necessary research monies to fund the development of better control technologies. Money spent on bed bug research now will save on considerable fiscal impacts in the future.

It was suspected early in the resurgence, that the high number of treatment failures probably indicated that modern bed bug strains were resistant to the main products being employed, the pyrethroids. Circumstantial evidence confirmed this when bugs were found walking on permethrin dust in 2004; a sample was collected and this strain was subsequently maintained as a laboratory colony. Later insecticide resistant profiling, comparing a modern strain with a susceptible old strain, revealed very high levels of resistance to the pyrethroids and, to a lesser extent, the carbamates [19,20]. The challenge this posed to the pest management industry was that most products registered for bed bug control in Australia before 2010 were based on the pyrethroids. There was also one registered carbamate (bendiocarb) and two organophosphates (diazinon and pirimiphos methyl), but no silicate or insect growth regulator [11]. While we have found no insecticide resistance to the organophosphates [19] the associated odor means that most responsible pest managers have been reluctant to use these products. In fact one hotelier recently complained to the senior author that the smell could still be detected in an organophosphate treated room one year after the initial application of insecticide.

Through the early years of the 21st Century the spread of bed bugs through accommodation complexes was well documented in Australia. For example, in one staff accommodation complex of 320 rooms, bed bugs were first detected in May 2003 [11]. As a result of poor pest treatment procedures by pest managers unfamiliar with the challenge of treating modern bed bug strains, the infestation rapidly spread. By June 2005, some 20% of the rooms became infested before total eradication was achieved by an appropriately trained pest management company. In a three star inner city 110 room hotel in Sydney, bed bugs were first treated (again quite poorly) during February 2006 [21]. In less than one year 38 rooms had evidence of activity. In a student college attached to a major university, three rooms were initially identified as being infested in October 2006. To save costs, the management of the college told maintenance staff to undertake control, which was attempted using cockroach bombs. Subsequently 33% of the rooms (14/42) became infested before an experienced pest manager was contracted [22].

Situations such as these are not isolated occurrences and provide insights into the factors behind the bed bug resurgence. Typically the most quoted reasons for the resurgence are insecticide resistance and increases in international travel. Clearly, resistance was the key trigger for the resurgence, and the spread of resistant bed bugs was aided by international travel. However, neither of these factors alone can explain the magnitude of the resurgence. The evidence above suggests that poor pest management practice has been a major contributing factor to the degree of the resurgence. Poor pest management can be directly linked to inadequate training of technicians in controlling insecticide resistant bed bugs. This is compounded by the lack of industry standards which set the benchmark for quality control.

By early in the 21st century, it became evident that bed bugs were becoming increasingly problematic in Australia, which was confirmed by the survey in 2006. Urgent strategies were thus required to curb the increase and the associated impacts. The immediate solution was to develop a pest management industry standard, called: ‘*A Code of Practice for the Control of Bed Bug Infestations in Australia*’ [13].

## 3. The Bed Bug Code of Practice

The Code of Practice for the Control of Bed Bug Infestations in Australia (herein designated the ‘Code’) was developed under the auspices of the Australian Environmental Pest Managers Association (AEPMA), the peak industry body for pest controllers. The concept of a bed bug industry standard was first outlined at the 2005 annual AEPMA conference and, at this meeting a Working Party (WP) was established. The composition and operation of the WP are described later. Having recently produced a 69 page article on bed bugs for the pest management industry [23], the senior author of this paper (SLD) was invited to be the Coordinator and Principal Editor of the ‘Code’. The 69 page article formed the basis of the ‘Code’ and, in 2006 a draft version of the ‘Code’ was released for public comment [24].

As noted above, the aim of the ‘Code’ was to encourage and promote the highest possible quality of pest management practice (*i.e.*, ‘best practice’), both in terms of the management of active infestations and also of potential infestations. For the purposes of the Code, ‘best practice’ was defined on the basis of adopting management tools for which there was evidence of efficacy through publications or common practice. In many cases where information was lacking for a particular technology, members of the WP have undertaken research to determine what constituted ‘best practice’. Only those technologies where there is demonstrable and/or independent evidence of efficacy are included in the ‘Code’. As bed bug research is continuing, highly dynamic and numerous control technologies are coming into the marketplace, the ‘Code’ is reviewed annually to ensure that it maintains ‘best practice’. To date, there have been six versions (*i.e.*, updates) of the ‘Code’ and three published ‘editions’.

The ‘Code’ now forms the basis of educational and training programs in bed bug management in Australia. It is envisaged that minimizing poor pest control will reduce both the health and financial impacts of bed bugs, and potentially lead to a halt and reversal of the resurgence. To encourage its use, the ‘Code’ has been made freely available from a dedicated web site [25]. It is a comprehensive and extensive document intended as a reference document from which other more focused articles can be produced.

Beyond the appropriate training in bed bug management, Codes of Practice can offer other benefits:

### Consumer Protection

Over recent years the market place has been flooded with devices that claim to either detect bed bugs or to control them. The quick push for profits, with almost no legislative oversight, has meant that quality independent efficacy data is often lacking. Many devices are even conceptually flawed; for example, the use of permethrin impregnated fabrics (our Sydney bed bug strain requires a lethal dose of 1.4 million times that of the susceptible strain with this insecticide [19]) and high pressure devices claiming to control bed bugs that tend to blow the insects about non-lethally, potentially spreading an infestation. The fact is that every device and insecticide used against bed bugs has limitations; the simple evidence for this is that bed bug resurgence is yet to abate. These limitations need to be understood by the end users. Thus both pest managers and their clients require protection against questionable products, and knowledge of a product's limitations. The Australian ‘Code’ only includes products, devices and management processes where there is evidence of efficacy. When listed, the limitations of the management tool or processes are discussed. This is in contrast to a number of bed bug procedural guides that attempt to list every available technology. Without providing the information on product limitations, such guides may actually contribute to the bed bug resurgence.

The ‘Code’ also helps to protect consumers against bad pest control. It has become the *de facto* industry standard. Failure of a pest manager to follow the ‘Code’, which results in treatment failure, may result in him or her being held liable unless written agreement not to follow the code is obtained from the client. Similarly, the ‘Code’ can protect pest managers from bad clients; if asked to undertake a quick spray for minimal money, the pest manager can refer to the inadequacies of such a treatment within the ‘Code’.

### Cost Effectiveness

Most strategies to combat the bed bug resurgence are both costly and take time to yield the benefits. For example, a research grant in Australia in the order of $100,000 would only employ one scientist for one year and any resulting publication would have limited scope and take time to produce. In recent years in the USA, a number of independent groups have developed bed bug procedural guidelines [26–33]. These take time and money to produce, and often contain broadly similar information. The ongoing ‘re-invention’ of identical information is a waste of limited public resources. A key industry Code of Practice, which can be used by all stakeholders is much more cost effective (the Australian ‘Code’ was estimated to cost around $15,000 in real terms) and can be produced in a relatively short period of time, providing immediate benefits.

### Protecting Health and the Environment

The extreme difficulty in achieving eradication with the current range of pyrethroids has led to the gross misuse of insecticidal products. In the Australian bed bug survey mentioned above, many (12.4%) pest managers reported the use of unregistered insecticides. One of the authors was told of an anecdotal report of a pest manager in the Middle East who used phosphine gas against bed bugs in an apartment complex, which resulted in the death of two children in an adjoining unit. The general public often goes to desperate attempts to rid themselves of bed bugs, in one case this led to a fire as a result of a tenant using rubbing alcohol as an insecticide [34]. An industry standard can provide the most appropriate means of safely achieving eradication.

### Assist Legislatures

In the US, for example, a number of legislative bodies are considering or undertaking laws, statutes and/or ordinances in relation to bed bugs [35]. The presence of an industry standard can help ensure legislation conforms to ‘best practice’.

As stated above, the principal aim of the Australian ‘Code’ is education in ‘best practice’, and current best practice involves combination of many factors—each of which contributes to control. This is true ‘Integrated Pest Management’ or IPM. The ‘Code’ is targeted towards anyone who is involved in the eradication of bed bugs, who may be impacted by bed bugs, who has responsibility of enforcing compliance, or may be in a position of inadvertently spreading bed bugs (e.g., used furniture sellers and linen contractors). The ‘Code’ aims to encourage communication, especially between the pest manager and client. Invariably, in managing an infestation, there are duties that the client must perform. Eradication is now seen as a cooperative effort between the pest manager and client in utilizing all appropriate means of control. This can be regarded as ‘Cooperative IPM’.

Broad acceptance of the ‘Code’ was a key objective. This has been achieved through widespread industry consultation, and a highly transparent and accountable approach to its development. Each time a draft is released, it is placed on the bed bug website for feedback for a minimum of six weeks and submissions sought from various stakeholders. All submissions are considered by the WP and recorded onto the bed bug web site. If a submission is rejected, the reasons for doing so are noted.

There are many aspects covered within the ‘Code’, some of the most salient sections include: education and training (of pest managers and those in the industries of providing accommodation), occupational health and safety, how to choose a pest manager with experience in bed bug management, bed bug identification, how to prepare and plan for inspections and treatments, inspection procedures, high risk factors that can make eradication more challenging, the need for pest managers to have a management plan, treatment processes (both non-chemical and chemical methodologies), post treatment procedures, prevention measures (which is really about harm minimization), and situational control. Included are various checklists to aid pest managers with service calls, suppliers of bed bug products and contacts, and a list of currently registered insecticides.

The international situation with the bed bug resurgence means that even if ‘best practice’ is largely conducted in one country and the bed bug problem abates, then the reintroduction of bugs from nations that do not have pest management standards is likely to occur. For this reason, the WP decided that the Australian ‘Code’ would be made freely available to any pest management association to help encourage ‘best practice’ worldwide. The first edition of the ‘Code’ was adopted by the Pest Management Association of New Zealand and the Italian pest management association (Associazione Nazionale delle Imprese di Disinfectazione [13]). More recently a registered charity in Europe was established to encourage “information, training and advice on prevention and treatment of Bed Bugs” within the whole of that continent. The Bed Bug Foundation [36] has now developed a comprehensive industry standard for Europe, with the first draft released in March 2011 for public comment [37]. This standard was based on the Australian ‘Code’. Other nations are now realizing that industry standards are essential to combat the bed bug resurgence and it is encouraging that in the US the National Pest Management Association has just developed a ‘best management practices’ document [38]. The presence of independently produced codes of practice means that each group can cooperate to further develop and refine ‘best practice’ in bed bug management.

One of the challenges has been to objectively determine if the ‘Code’ is achieving the aims of promoting better education in bed bug management, and if this has flowed on to reduce the numbers of infestations. In early 2010, another bed bug survey of pest managers was conducted by the authors and, superficially, the results indicated a continuing rise in bed bug infestations [39]. However, there were few respondents to the second survey and most of the infestations reported were from members of the WP for the ‘Code’ who have become recognized experts and sought after for bed bug management business. Thus the second survey is biased by the existence of the ‘Code’ and it will probably become increasingly difficult to obtain accurate data on bed bug infestations into the future. Anecdotal evidence suggests the ‘Code’ has been beneficial. The authors of this report are hearing of fewer treatment failures and less inappropriate advice given to clients, while massive infestations involving tens of thousands of bed bugs have not been seen since 2008. A number of pest managers have stated to the authors that the ‘Code’ has aided client communication, helped streamline the treatment processes and, if clients adhere to the processes as outlined with the ‘Code’, then this more likely results in a successful treatment. One insecticide manufacturer has commented that as the ‘Code’ promotes integrated pest management and prolongs the market life of products, thereby helping to maintain long term profits for the company. Also, companies have begun referencing the ‘Code’ on labels of products being registered against bed bugs. Thus it appears that the objectives of the ‘Code’ are becoming realized.

## 4. The Bed Bug Code of Practice Working Party

The WP responsible for the development of the ‘Code’ was first established in 2005. The group consisted of representatives of AEPMA, recognized industry leaders in pest management (including practicing technicians), entomologists, and employees of an insecticide manufacturer.

To ensure proper conduct of the WP, a document; ‘*Guidelines for the Establishment and Management of AEPMA Code-of-Practice Working Parties*’ [40] was developed and endorsed by the Board of AEPMA. This document defines the role and logistics of the WP, the membership criteria and probity requirements, and is being used for all AEPMA Code of Practice working parties. Pest management associations are welcome to adopt the Guidelines, which are freely available for download [41].

Having developed and published the ‘Code’, the main role of the WP now is to regularly review it, advise on appropriate training and accreditation, and highlight research requirements and knowledge deficiencies. Review of the ‘Code’ must be undertaken at least every 18 months otherwise it is considered out of date and no longer ‘best practice’. Similarly, if the WP becomes inactive for 12 months then it will be declared disestablished. In order to continually update the knowledge of the WP, members circulate by email through the WP Chair, articles and information on bed bugs gleaned from sources appropriate to their particular roles. This enables members to consider if new materials, products or control methods warrant inclusion in subsequent editions of the ‘Code’. Between 4 December 2008 and 31 January 2011, 265 such documents were circulated and these included formal and industry publications, news items from pest management magazines and circulars, product information, conference papers and notes, media releases and reports, possible amendments to the ‘Code’, and various other materials. Typically the WP meets face to face twice a year, where aspects of the ‘Code’ are reviewed. Other standing items on the meeting include: WP membership review, unusual case studies, bed bug training requirements, research and product updates, new publications, and overseas activities and collaborations. The Chair of the WP reports the activities of the WP to the AEPMA Board annually in accordance with the Guidelines.

## 5. A Bed Bug Management Policy for Accommodation Providers

The ‘Code’ was developed with the aim of defining the procedures necessary to successfully eradicate bed bug infestations wherever they may occur. However, the management of specific processes required by those in the industries of providing accommodation was (arguably) not succinctly addressed. The accommodation industry is in reality a diverse group covering those in hospitality (hotels, motels, backpacker hostels, resorts, caravan parks, bed and breakfasts, farm stays, *etc.*), landlords, student and staff accommodation, public and community housing, aged care facilities, transport, campervans and motor homes, and hospitals, to name a few. Such organizations have a duty of care to provide facilities that are safe to clients and free of vermin.

In recent years, especially within the United States, there has been a spate of litigation against accommodation providers (particularly against hospitality groups) as a result of guests being attacked by bed bugs. Many of these cases have become very high profile, being widely reported in the media, and a quick search on the internet will reveal many such examples. However, it appears that very few make it to trial and presumably settled out of court. It is believed that the reason for this is that the accommodation providers have been unable to demonstrate ‘due diligence’, namely to show that they ‘took all reasonable steps’ to minimize the risk of bed bug infestations and to eliminate them in a timely manner when they occurred. In all probability, such cases have been settled out of court to avoid more serious fiscal (and public) damage.

Despite the diversity of accommodation available, there is much in common about how such groups can process manage bed bug infestations. Thus in order to compliment the ‘Code’, a ‘*Bed Bug Management Policy for Accommodation Providers*’ was developed in 2010 [14].

This document is ostensibly a policy and procedural guide, and has been subject to the same public scrutiny, review and developmental processes as the ‘Code’, and is also freely available for download [25]. The policy is supplementary to and regularly cross references the ‘Code’. The main sections of the policy include:

### Defining Responsibilities

The responsibilities of the main stakeholders in relation to bed bug management are defined. These are the facilities management, the tenant (or client) and the pest manager.

### Education and Training

This details the training aids required to educate staff, tenants and external agencies in how to identify and manage potential or active bed bug infestations.

### Documentation

The documentation required for the process of bed bug management includes policies and procedures, records of bed bug mitigation and the detailed recording of infestations and treatments. Documentation is essential for providing evidence that bed bug management has been undertaken in a timely and appropriate manner. A database with proposed fields is provided.

### Occupational Health and Safety (OH&S)

The emphasis is on OH&S for staff who may have to deal with bed bugs, and includes information how staff should conduct themselves in an infested room, personal protection equipment, required disinsection (freeing an area of insects [42]) facilities, and the need to keep records of material safety and data sheets or product labels for any insecticide used.

### Eradication Processes

A brief overview of the salient features of bed bug eradication as in accordance with the ‘Code’ is provided.

### Preventative Measures

Like the ‘Code’, ‘Preventative Measures’ are about harm minimization, thus aim to reduce the risk of infestations and to minimize their effects when bed bugs do occur.

### Media

Just as every large organization has a policy that dictates who speaks with and what is spoken to the media, an individual familiar with bed bugs should be the designated media contact officer.

The reality is that it is impossible to definitely prevent bed bug infestations with current pest control technologies and it is likely that many tenants/guests will fall victim to bed bugs. Having a bed bug management policy which is actively followed could provide evidence of ‘due diligence’ and minimize litigation risks, and also help to reduce associated adverse publicity. Ensuring that due processes are undertaken will reduce the risk of bed bugs spreading and minimize overall management costs.

Ultimately, a bed bug management policy is about saving money for an organization, both in terms of current monies but also potential future earnings. A Bed Bug Management Policy thus becomes a brand protection tool.

## 6. Education and Training

Education of all stakeholders in best practice management is a key factor in minimizing potential bed bug impacts. Like other strategies, education needs to be multifaceted, encompass different forms of delivery and often needs to be tailored to a specific group for their particular requirements. In Australia, the authors and WP members have conducted bed bug training courses, provided numerous lectures on bed bugs, published many articles, and have a dedicated bed bug web site.

A bed bug training course was developed in 2009 by the WP, utilizing a curriculum based on the ‘Code’. In doing this, it was possible to ensure that the course provided a coordinated approach to bed bug management rather than being a set of disparate lectures. The course was made generic to appeal to anyone who has to deal with bed bug eradication. Pest managers and the hospitality industries were encouraged to attend so that both groups could share their experience and challenges. All attendees received a copy of the ‘Code’ as well as comprehensive course notes [43] that complimented the ‘Code’. The course notes contained information on bed bug biology, health and financial impacts, details on the resurgence, how bed bugs are spread, an overview of control, details of insecticide efficacy studies, case studies on challenging infestations, comprehensive photographs from infestations, and actual bed bug samples for reference purposes. Pest managers who attended the course were eligible for continuing professional development points as part of the local accreditation system. To assist those with bed bug infestations in finding trained operators, all pests managers who successfully completed the course were listed on the ‘Code’ web site. All attendees were issued with comprehensive feedback forms, which were later reviewed by the WP to further improve the course. Presently this course is a one day event that aims to ‘train the trainers’. A much longer course is in development aimed at pest management technicians.

Despite the ‘Code’ being first developed in 2006 it was three years before the first bed bug course was held. This was due to a lack of support, particularly from the hospitality industries, which is quite surprising since they are the group who would most benefit from improved training of pest technicians. The generosity of a WP member, who provided initial financial backing, enabled the course to be held, though ultimately it covered its costs.

Over the last seven years the authors have given numerous lectures (>90) on bed bug management to various groups, this has included; pest managers (31 lectures), low income housing providers (17), hospitality and tourism groups (10), environmental health officers (9), medical scientists (8), professional entomologists (7), medical staff (4), elite sporting institutions (1), student accommodation (1), a bedding and mattress exhibition (1), local government politicians (1), community forums (1), and animal technicians (1).

Similarly, many articles (56 as of January 2011) on bed bugs have been published by the authors. These have not so much been aimed at academic impact, as at societal impact, so they have largely appeared in specific industry orientated publications; for example, publications for pest managers (32 articles), the hospitality industry (8), professional scientific journals (6), executive housekeepers (5), student accommodation (2), hotel engineers (2), and medical journals (1).

The bed bug web site [25] was developed for the ‘Code’. It now holds all six versions along with the public submissions, the Bed Bug Management Policy for Accommodation Providers, the list of pest managers trained in bed bug management, a bed bug fact sheet and articles. All information on this site is freely available and typically this web site ranks highest on Google Australia for dedicated bed bug web sites.

## 7. Research

The development of the ‘Code’ highlighted many deficiencies in our knowledge on bed bug management. At the time of the first draft in 2006, there were no recent papers on insecticide efficacy on modern field strains from anywhere in the world and certainly none from Australia. There was little information available on non-chemical means of control such as the appropriate use of vacuuming, or steam, while old pest controller tales of the use of black plastic for the thermal control of bed bugs on mattresses still abounded, despite the lack of scientific evidence confirming efficacy. As a result, various members of the Working Party undertook testing on a range of non-chemical control tools, profiling the insecticide resistance of modern stains and testing the efficacy of various insecticide products. The results have been used to refine and evolve the ‘Code’, and presented at various conferences and/or published in industry journals or scientific forums [11,19,20,44–48].

Most of the research has come about through the collaboration of health scientists (Medical Entomology, Westmead Hospital) and the Australian branch of an international pest management firm, Ecolab Pest Elimination. This collaboration has provided immediate benefits to the company; the insecticide efficacy testing has demonstrated which products are most effective and this in turn has led to the modifications of bed bug management protocols. The company is now seen as one of the leaders in bed bug management and this has resulted in an expanded customer base. Such benefits should encourage other companies to undertake partnerships of this kind.

## 8. Conclusions and the Future

The above strategies to combat bed bugs in Australia have been achieved without any specific financial support. The WP members have funded their own attendance at meetings, workshops and training courses, often at considerable expense. Fortunately this has been offset to a degree by tangible benefits for the pest managers; the information circulated through the WP has led to increased knowledge and the members have become recognized bed bug experts in their respective states, leading to improved business opportunities.

Bed bugs however remain problematic and Australia faces a number of challenges in the future. Hospitality groups have failed to become involved in combating the rise of bed bugs and such short sightedness has clearly damaged their own industry, and probably contributed to the resurgence. Many are still not even aware (or admit!) there is a bed bug resurgence until the insect impacts upon them or such impact becomes public knowledge. Yet such groups need to be educated on the issue and encouraged in the long term battle against bed bugs.

There are still pest managers who are not aware that the ‘Code’ exists, despite the myriad of lectures and publications presented to the industry. There is a subset of the pest management industry that does not undertake any professional development and getting the ‘Code’ known to this group is difficult (these are often where control failures still occur). It is encouraging that most insecticide manufacturers are now including on their new product labels a recommendation that the ‘Code’ is followed. This should further help raise awareness of the ‘Code’.

The level of awareness of bed bugs in the general community is still low. Most people, including intelligent and well-informed individuals, still do not know that bed bugs are a serious problem, why they are a problem, what bed bugs look like, how someone would know if they have bed bugs and what they should do if they find an infestation. Much of the material on bed bugs in the public domain is ‘tainted’ as the information presented is about selling something and thus many people have become skeptical about the whole problem. The media especially has a tendency to trivialize the bed bug problem in order to make it more sensational (how often does one hear on television or see in print, “night, night, sleep tight, don't let the bed bugs bite”). Trivializing actually ‘detaches’ people from the issue when what is really required is for them to ‘relate’ to it.

It is clear that no magical ‘silver bullet’ to control bed bugs will appear in the near future. The cost of insecticide development means that specific products are unlikely to be produced for bed bugs, while most non-chemical means of control will, at best, be capable of reducing the bed population in an infestation but not eliminating it. Thus the strategies of having industry pest management standards, and providing education of ‘best practice’ based on Cooperative Integrated Pest Management, will remain the key means by which bed bugs will be combated into the future.

Bed bugs are an international problem with significant commonality now recognized in all populations worldwide. Hence international solutions are required. We need to share the results of research and the experiences of pest managers, and we need international collaboration on evolving industry standards. The ultimate defeat of the current bed bug resurgence will not be achieved by one group or country; it will be from the combined efforts of many.
